# Association between waist-height ratio and hypertension: results from the lookup 8+ study

**DOI:** 10.3389/fmed.2026.1975590

**Published:** 2026-10-21

**Authors:** Federica D’Ignazio, Giulia Giordano, Antonella Risoli, Luca Mastrantoni, Riccardo Calvani, Sara Salini, Andrea Russo, Vincenzo Galluzzo, Francesca Ciciarello, Francesco Landi

**Affiliations:** 1 Department of Geriatrics, Fondazione Policlinico Universitario IRCCS A. Gemelli, Rome, Italy; 2 Università Cattolica del Sacro Cuore, Rome, Italy; 3 Comprehensive Cancer Center, Fondazione Policlinico Universitario IRCCS A. Gemelli, Rome, Italy

**Keywords:** cardiovascular health, cardiovascular screening, central adiposity, hypertension, waist-to-height ratio

## Abstract

**Introduction:**

Hypertension is the leading cardiovascular disorder, with central adiposity among its key modifiable determinants. The waist-height ratio (WHtR) has been proposed as an index of central adiposity, yet its association with hypertension and its comparative utility for hypertension screening remains incompletely defined. This study investigated association between WHtR and hypertension and compared its discriminatory performance with body mass index (BMI) and waist-hip ratio (WHR).

**Methods:**

A total of 14,415 community-dwelling adults were included in the analysis. Hypertension was defined as systolic blood pressure (SBP) ≥140 mmHg and/or diastolic blood pressure (DBP) ≥90 mmHg, and/or current antihypertensive therapy. Binomial and multivariate generalized linear models (GLM) assessed association between WHtR and blood pressure, adjusting for sex, age, and five health metrics. ROC curve analyses with DeLong pairwise comparisons evaluated discriminatory performance, stratified by sex.

**Results:**

Abnormal WHtR (*n* = 8,731) was significantly associated with higher SBP (130 vs. 120 mmHg; *p* < 0.001) and DBP (80 vs. 70 mmHg; *p* < 0.001), and with greater hypertension prevalence, particularly in older and male participants. Sex-adjusted GLM showed abnormal WHtR negatively associated with normal blood pressure (OR 0.25; *p* < 0.001), persisting after full adjustment (OR 0.44; *p* < 0.001). Continuous GLM confirmed positive associations with SBP (*β* = 6.11 per 0.1-unit increment; *p* < 0.001) and DBP (*β* = 2.36; *p* < 0.001). WHtR demonstrated superior discriminatory accuracy over BMI and WHR overall (AUC 0.7285 vs. 0.6901 vs. 0.6869) and across both sexes, confirmed by DeLong comparisons.

**Conclusion:**

WHtR outperforms BMI and WHR in identifying individuals with hypertension across both sexes, supporting its evaluation for cardiovascular screening in community settings.

## 1 Introduction

Hypertension is the most widespread cardiovascular disorder: the World Health Organization estimates it affects 1.28 billion adults aged 30–79, two-thirds in low-middle-income countries (1). In 2021 hypertension was the most prevalent metabolic diseases, showing great impact on global disease burden (2). Adults with blood pressure above the threshold value of ≥140/90 mmHg are at increased risk of cardiovascular diseases, with an estimated 10-year risk of fatal and non-fatal cardiovascular events ≥10% (3).

The 2024 ESC guidelines stress addressing modifiable lifestyle factors (including diet, body weight, and physical activity) since obesity and central adiposity are central to hypertension aetiology and amenable to diet and exercise interventions (3). Recent evidence underlined the role of central adiposity in increasing cardiovascular diseases (CVD), stroke, and cardiovascular (CV) death risk (4). Anthropometric indices may help identify individuals at higher hypertension risk, enabling earlier intervention (3, 5). Widely used measures include body mass index (BMI), waist-hip ratio (WHR) and waist-to-height ratio (WHtR). The BMI is the most commonly used measure of central adiposity, still it carries several limitations in epidemiological applications, due to thresholds reference variability among different ethnic groups, inadequacy in reflecting age-related body changes and sex-specific difference in body composition, and may potentially not detect weight gain nature, depending on increased fat mass or muscle mass (6). The WHtR represents a simple and low-cost tool, with a defined cutoff at 0.5 for both sexes, robust across different ethnic populations (5). This threshold was selected as reference to define if patient has increased central adiposity and was validated both in younger subjects and adults (7).Growing interest is emerging on this index ‘potentiality in clinical applicability, as confirmed by recent works on the topic (8). Compared to BMI, waist circumference (WC), and WHR, WHtR performed as a more specific measure in abdominal adiposity assessment and several studies confirming its superiority in identifying cardiometabolic risk, for both sexes (7, 9, 10). Evidence confirmed that both abnormal WHR and WHtR are associated with cardiometabolic diseases, type 2 diabetes, CVD and hypertension (5, 7, 11–13); National Institute for Health and Care Excellence (14, 15). NICE included WHtR in 2023 obesity assessment guidelines as a measure of central adiposity (16). A meta-analysis by Jayedi et al. found summary relative risks (RR) of 1.49 [95% confidence interval (CI): 1.41–1.58; *I*^2^ = 97.4%, *n* = 50] for a 5-unit increment in BMI, RR 1.27 (95% CI: 1.15–1.39; *I*^2^ = 95.0%, *n* = 14) for a 10 cm increment in WC, RR 1.16 (95% CI: 1.09–1.23; *I*^2^ = 77.8%, *n* = 5) for weight gain (corresponding to a 1-unit increment in BMI), and RR 1.37 (95%CI: 1.24–1.51; *I*^2^ = 76.4%, *n* = 8) and 1.74 (95% CI: 1.35–2.13; *I*^2^ = 58.9%, *n* = 4) for a 0.1-unit increment in WHR and WHtR respectively (17). This confirms that hypertension risk grows continuously with increasing anthropometric measures, along with weight gain, with WHtR showing the highest RR.

Considering the increasing interest on WHtR in cardiovascular screening for its potentiality as a simple and effective anthropometric measure for identifying hypertension, and the lack of evidence from community-dwelling adults large cohorts, we aimed to investigate association between WHtR and hypertension in the large population of the LookUp 8+ study.

## 2 Methods

This study was based on a retrospective analysis on cross-sectional data from the Lookup 8+ study, an ongoing initiative supported by the Department of Geriatrics of the Catholic University of the Sacred Heart and the “Agostino Gemelli” University Hospital Foundation IRCCS in Rome, Italy. The project was developed to promote healthy lifestyle adoption and increase population awareness about preventable risk factors for chronic diseases. The study protocol was approved by the Ethics Committee of the Catholic University of the Sacred Heart (Protocol #A.1220/CE/2011). A full description of the protocol is available elsewhere (18–20). Prior to enrolment, a written informed consent was obtained from all participants. The present manuscript was prepared in accordance with the Strengthening the Reporting of Observational Studies in Epidemiology (STROBE) reporting guidelines for observational studies (21). The STROBE checklist can be found in Supplemental Material.

### 2.1 Sample selection

Between June 1st 2015 and December 1st 2025, a total of 24,445 individuals were enrolled through recruitment activities and community events in public settings such as large metropolitan areas and major Italian islands. Eligibility criteria included to be at least 18 years old and provide a written informed consent. Exclusion criteria were a reported current pregnancy, being unable to complete physical performance assessments, declining capillary blood sampling for the measurement of total cholesterol or blood glucose, being unable or unwilling to provide informed consent. The present analysis was restricted to individuals who underwent a complete anthropometric assessment, including the measurement of both waist circumference, and height.

### 2.2 Data collection

Participants completed a brief questionnaire on lifestyle habits and underwent anthropometric assessment, capillary blood sampling, blood pressure measurement, and physical performance evaluations. Smoking behaviour was originally classified into three categories: current smokers, defined as individuals who had smoked at least 100 cigarettes during their lifetime and were still smoking at the time of assessment; never smokers, who had never smoked or had smoked fewer than 100 cigarettes overall; and former smokers, defined as those who had smoked at least 100 cigarettes but had quit at least 28 days prior to the interview. For the statistical purposes of this study, smoking status was dichotomized into smokers and non-smokers (including never or former smokers) (22, 23). A healthy dietary pattern was defined as daily consumption of at least 3 portions of fruits and/or vegetables. Estimates of daily fruit and vegetable intake were derived using standard reference tables developed by the Italian Society of Human Nutrition (SINU) for the Italian population. In the analyses, fruit and vegetable intake was used as a surrogate marker of overall dietary quality, serving as an indirect indicator of adherence to a healthy nutritional pattern (24). Physical activity was defined as exercise for at least 30 min per session, at least twice per week, during the previous year. The type of activity performed was categorized as low-intensity walking, aerobic exercise (running, cycling, or swimming), or strength training, with or without stretching. For analytical purposes of this study, participants were subsequently grouped as physically active or physically inactive, referring to previous validated categorization on this cross-sectional population (25). The total blood cholesterol was measured using a portable reflectometric analyser with disposable test strips (MultiCare-In, Biomedical Systems International srl, Florence, Italy) (26). Capillary blood glucose levels were obtained using disposable strips based on a detection system with the MultiCare-In portable device. In accordance with the American Diabetes Association guidelines, participants were classified as having diabetes if they reported a previous diagnosis of diabetes, had a fasting plasma glucose level ≥126 mg/dL, or presented with a random blood glucose concentration ≥200 mg/dL (27).

### 2.3 The five health metrics

Due to growing interest in cardiovascular health, the American Heart Association (AHA) developed the Life's Essential 8 framework (28): it consists of 8 potentially modifiable health metrics: diet quality, physical activity, smoking status, body-mass index, total cholesterol, fasting blood glucose, blood pressure, and sleep quality. Each component can be scored on a continuous scale ranging 0–100, or can be combined in a composite score, with higher values indicating better overall cardiovascular health. We selected 5 out of the total 8 health metrics for adjustment in our analysis, including absence of smoke habits (“Smoke HM”), healthy diet (“Diet HM”), physical activity (“PA HM”), normal total blood cholesterol (“Cholesterol HM”), and normal blood glucose values (“Glucose HM”), and categorized them as absent or present.

### 2.4 Blood pressure measurement

Blood pressure was measured with a manual sphygmomanometer in accordance with European guideline recommendations (3). For all participants, after a 5 min seated rest, with three consecutive measurements taken at 30 s intervals; the mean of these readings was used for analysis. Cuff size was selected based on individual arm circumference to ensure measurement accuracy. All assessments were conducted in a quiet, dedicated room under standardized environmental conditions. Blood pressure levels were classified as <120/80 mmHg in untreated individuals, 120/80–139/89 mmHg (or controlled with treatment), and ≥140/90 mmHg. Information on the use of antihypertensive medications was also collected. Hypertension was defined by a mean systolic blood pressure (SBP) of at least 140 mmHg and/or a mean diastolic blood pressure (DBP) of at least 90 mmHg, and/or current use of antihypertensive therapy (3, 19, 20, 23).

### 2.5 Anthropometric measurements and waist-height ratio assessment

We assessed anthropometric indices with participants wearing thin clothing and with no shoes on. Body weight was measured using an analog scale, while height was determined using a stadiometer. Body mass index (BMI) was calculated by dividing weight (in kilograms) by the square of height (in meters). Waist and hip circumferences were assessed, and WHtR was calculated according to NICE guidelines (National Institute for Health and Care Excellence (14) by dividing waist circumference by height measurement. Anthropometric measurements were assessed using a non-stretchable pliable plastic tape. WHtR is calculated as the ratio of waist circumference to standing height (10); National Institute for Health and Care Excellence (14). Waist circumference is obtained at the midpoint between the inferior margin of the last rib and the iliac crest, assessed during normal expiration (6). The measuring tape was placed with a comfortable tightness, ensuring it did not apply force on the skin (29). A WHtR value ≥0.5 is considered as abnormal, suggesting increased central adiposity and increased cardiovascular health risk (30).

### 2.6 Statistical analysis

Study participants’ characteristics were described by WHtR group. Categorical variables are reported as absolute numbers and percentages, while continuous variables are expressed as median and interquartile range (IQR). Differences in categorical variables between groups were evaluated using the Pearson chi-squared test, while differences in continuous variables were assessed using Student's *t*-test or the Mann–Whitney *U*-test, as appropriate. In all analyses, statistical significance was defined at *p* < 0.05. In this study we considered both WHtR as a continuous and a dichotomous variable, categorized as abnormal when ≥0.5, accordingly to the analysis. In all categorical analyses, “normal WHtR”, “female sex”, and the absence of the respective health metric risk factor (score = 0, indicating an altered health metric) were established as the reference categories, meaning that a score of 1 represents the healthy status for each metric. To assess the association between WHtR and a normal blood pressure, a binomial generalized linear model (GLM) analysis was performed, along with a GLM multivariate analysis with systolic blood pressure (SBP) and diastolic blood pressure (DBP) as dependent variables and WHtR as the independent variable. Results are reported as coefficients with relative 95% confidence intervals (95% CI) and corresponding *p*-values. The assumption of normality was verified by reviewing histograms of the dependent variable. Both binomial and multivariate GLM analyses were conducted as sex-adjusted and as multi-adjusted, including sex, age, and five health metrics (smoke habits, healthy diet, physical activity, total blood cholesterol, and blood glucose). To evaluate the discriminatory performance of WHtR, BMI, and WHR for hypertension, receiver operating characteristic (ROC) curves were constructed for each anthropometric index. The area under the curve (AUC) was calculated with 95% CI, and sensitivity and specificity at the respective thresholds were determined. Pairwise comparisons between AUC values were performed using the DeLong nonparametric method (31). ROC analyses were then conducted separately for males and females. All analyses were performed using R Studio (vers. 4.5.2).

## 3 Results

A total of 14,415 subjects had no missing data in all variables of interest and were therefore included in the analyses. A flow diagram with participants selection details is reported in Supplemental Material (Supplementary Figure S1). Median age was 57 years (IQR: 47.0–67.0), and males represented 45% of the cohort (*n* = 6,528). Advanced age was significantly associated with abnormal WHtR: participants in the abnormal WHtR group had a median age of 61 years (IQR: 53.0–71.0), compared with 50 years (IQR: 39.0–58.0) in those with normal WHtR (*p* < 0.001). Among 8,731 participants showing abnormal WHtR, 4,644 were males, confirming males’ tendency to exhibit greater central adiposity. In this cohort, abnormal WHtR was significantly associated with cardiovascular risk factors, including higher BMI (median 26.0, IQR: 24.2–28.4 kg/m^2^; *p* < 0.001), higher prevalence of altered glucose levels (12% vs. 4.4%; *p* < 0.001), reduced physical activity (44% vs. 28%; *p* < 0.001), lower adherence to a healthy diet (40% vs. 36%; *p* < 0.001), higher prevalence of hypercholesterolemia (54% vs. 46%; *p* < 0.01), and higher prevalence of hypertension (67% vs. 32%; *p* < 0.001). The association extended to both SBP (median 130 mmHg, IQR: 120–140 mmHg; *p* < 0.001) and DBP (median 80 mmHg, IQR: 70–80 mmHg; *p* < 0.001). All descriptive statistics are reported in Table 1.

**Table 1:** Descriptive characteristics of the study sample.

Characteristic	Overall (*N* = 14,415)	Normal (*N* = 5,684)	Abnormal (*N* = 8,731)	*P* value^a^
Age	57 (47, 67)	50 (39, 58)	61 (53, 71)	<0.001
Sex				<0.001
Female	7,887 (55%)	3,800 (67%)	4,087 (47%)	
Male	6,528 (45%)	1,884 (33%)	4,644 (53%)	
Waist-Height Ratio (WHtR)	0.52 (0.47, 0.58)	0.45 (0.43, 0.48)	0.56 (0.53, 0.61)	<0.001
Waist-Hip Ratio (WHR)	0.88 (0.82, 0.94)	0.81 (0.77, 0.86)	0.92 (0.88, 0.97)	<0.001
BMI	24.2 (21.9, 26.9)	21.6 (20.2, 23.1)	26.0 (24.2, 28.4)	<0.001
Systolic Blood Pressure (SBP)	120 (110, 135)	120 (110, 130)	130 (120, 140)	<0.001
Diastolic Blood Pressure (DBP)	75 (70, 80)	70 (70, 80)	80 (70, 80)	<0.001
Blood Pressure (HM)				<0.001
Altered (0)	7,718 (54%)	1,839 (32%)	5,879 (67%)	
Healthy (1)	6,697 (46%)	3,845 (68%)	2,852 (33%)	
Physical Activity (HM)				<0.001
Inactive (0)	5,437 (38%)	1,568 (28%)	3,869 (44%)	
Active (1)	8,978 (62%)	4,116 (72%)	4,862 (56%)	
Smoke (HM)				0.12
Smoker (0)	2,730 (19%)	1,112 (20%)	1,618 (19%)	
Non-Smoker (1)	11,685 (81%)	4,572 (80%)	7,113 (81%)	
Diet (HM)				<0.001
No Healthy Diet (0)	5,528 (38%)	2,045 (36%)	3,483 (40%)	
Healthy Diet (1)	8,887 (62%)	3,639 (64%)	5,248 (60%)	
Cholesterol (HM)				<0.001
Altered (0)	7,383 (51%)	2,631 (46%)	4,752 (54%)	
Healthy (1)	7,032 (49%)	3,053 (54%)	3,979 (46%)	
Glucose (HM)				<0.001
Altered (0)	1,342 (9.3%)	252 (4.4%)	1,090 (12%)	
Healthy (1)	13,073 (91%)	5,432 (96%)	7,641 (88%)	

Continuous variables are expressed as Median (IQR); categorical variables are expressed as *n* (%). Hypertension is defined by: mean systolic blood pressure ≥140 mmHg and/or a mean diastolic blood pressure ≥90 mmHg, and/or current use of antihypertensive therapy. Statistical significance is set at *p* value <0.05.

a Mann–Whitney *U*-test; Pearson's Chi-squared test.

WHtR, waist-height ratio; WHR, waist-hip ratio; BMI, body mass index; SBP, systolic blood pressure; DBP, diastolic blood pressure; HM, health metrics.

To evaluate the association between WHtR and a normal blood pressure profile, we performed two GLM binomial analysis, one adjusted for sex and a second including adjustment for sex, age and the five-health metrics (entitled from now on as “multi-adjusted” model). The sex-adjusted analysis showed odds ratio (OR) of 0.25 (95% CI: 0.24 to 0.27, *p* < 0.001) for abnormal WHtR, hinting that individuals with greater values of central adiposity presents 75% lower odds of maintaining a normal blood pressure profile, compared to those with normal WHtR. For sex, males exhibited OR of 0.55 (95% CI: 0.51 to 0.59, *p* < 0.001) compared to females. The multi-adjusted model reported OR of 0.44 (95% CI: 0.40 to 0.47, *p* < 0.001) for WHtR, confirming that even after controlling for age, sex, and the five-health metrics, an abnormal WHtR reduces the odds of having normal blood pressure by 56%. Among the five health metrics, Glucose (HM) emerged as significant, with OR of 1.47 (95% CI: 1.27 to 1.69, *p* < 0.001) suggesting that subjects with ideal fasting blood glucose levels had 47% higher odds of normal blood pressure compared to those with altered glucose. Age showed OR of 0.95 (95% CI: 0.95 to 0.95, *p* < 0.001) and sex reported OR of 0.46 (95% CI: 0.43 to 0.50, *p* < 0.001) (Table 2).

**Table 2:** Binomial generalized linear models.

Model 1			Model 2		
Characteristic	OR (95% CI)	*P* value	Characteristic	OR (95% CI)	*P* value
Sex			Sex		
Female	[Reference]		Female	[Reference]	
Male	0.55 (0.51–0.59)	<0.001	Male	0.46 (0.43–0.50)	<0.001
WHtR			WHtR		
Normal	[Reference]		Normal	[Reference]	
Abnormal	0.25 (0.24–0.27)	<0.001	Abnormal	0.44 (0.40–0.47)	<0.001
	–		Age	0.95 (0.95–0.95)	<0.001
	–		PA (HM)	1.08 (1.00–1.17)	0.062
	–		Smoke (HM)	1.04 (0.95–1.15)	0.368
	–		Diet (HM)	1.00 (0.93–1.09)	0.903
	–		Cholesterol (HM)	1.11 (1.03–1.20)	0.008
	–		Glucose (HM)	1.47 (1.27–1.69)	<0.001

Model 1: adjusted for sex. Model 2: with adjustment for sex, age and five health metrics. Hypertension is defined by: mean systolic blood pressure ≥140 mmHg and/or a mean diastolic blood pressure ≥90 mmHg, and/or current use of antihypertensive therapy. Statistical significance is set at *p* value < 0.05. OR, odds ratio; 95% CI, 95% confidence interval.

WHtR, waist-height ratio; PA, physical activity; HM, health metrics.

We then explored the association between WHtR and both SBP and DBP within a multivariate GLM analysis, both conducted as sex-adjusted and multi-adjusted. Our findings revealed a significant positive association between WHtR and SBP, with a β of 6.11 (95% CI: 5.79 to 6.44; *p* < 0.001) per 0.1 increase in WHtR, meaning that every 0.1-unit increase in the WHtR corresponds to an estimated 6 mmHg increase in SBP values. Male sex was independently associated with higher SBP, with β of 5.57 (95% CI: 5.07 to 6.07; *p* < 0.001) compared to females (Table 3a). In the multi-adjusted model, WHtR showed β 4.47 (95% CI: 4.10 to 4.83; *p* < 0.001) representing approximately 4 mmHg increase in the SBP for a 0.1 WHtR augmentation, while male sex reported β was 5.81 (95% CI: 5.30 to 6.31; *p* < 0.001) compared to females. Age was significantly associated with SBP, with β of 0.21 (95% CI: 0.19 to 0.23; *p* < 0.001), while none of the five-health metrics reported statistically significant association (Table 3a). In the sex-adjusted GLM multivariate analysis for DBP, a significant positive association was found with WHtR, with β of 2.36 (95% CI: 2.15 to 2.56; p: <0.001) per 0.1 increase in WHtR, representing a 2 mmHg increase in DBP for each 0.1 WHtR increment. Male sex was also independently associated with higher DBP, with β 3.15 (95% CI: 2.83 to 3.47; *p* < 0.001) compared to females (Table 3b). These findings were confirmed in multi-adjusted GLM analysis, where among the five-health metrics only Glucose (HM) emerged as significant, with β 1.51 (95% CI: 0.96 to 2.07, *p* < 0.001) (Table 3b).

**Table 3:** Multivariate generalized linear models for waist-height ratio and systolic and diastolic blood pressure.

Table 3a					
Model 1			Model 2		
Characteristic	β (95% CI)	*P* value	Characteristic	β (95% CI)	*P* value
Sex			Sex		
Female	[Reference]		Female	[Reference]	
Male	5.57 (5.07 to 6.07)	<0.001	Male	5.81 (5.30 to 6.31)	<0.001
WHtR^a^	6.11 (5.79 to 6.44)	<0.001	WHtR^a^	4.47 (4.10 to 4.83)	<0.001
	–		Age	0.21 (0.19 to 0.23)	<0.001
	–		PA (HM)	0.53 (0.01 to 1.05)	0.044
	–		Smoke (HM)	−0.19 (−0.81 to 0.44)	0.553
	–		Diet (HM)	0.17 (−0.35 to 0.68)	0.523
	–		Cholesterol (HM)	0.26 (−0.25 to 0.77)	0.314
	–		Glucose (HM)	0.67 (−0.19 to 1.52)	0.129

Table 3b					
Sex			Sex		
Female	[Reference]		Female	[Reference]	
Male	3.15 (2.83 to 3.47)	<0.001	Male	3.20 (2.87 to 3.53)	<0.001
WHtR^a^	2.36 (2.15 to 2.56)	<0.001	WHtR^a^	2.36 (2.12 to 2.59)	<0.001
			Age	0.01 (0.00 to 0.02)	0.103
	–		PA (HM)	−0.04 (−0.37 to 0.30)	0.820
	–		Smoke (HM)	0.01 (−0.40 to 0.41)	0.972
	–		Diet (HM)	−0.14 (−0.47 to 0.19)	0.394
	–		Cholesterol (HM)	−0.18 (−0.50 to 0.15)	0.290
	–		Glucose (HM)	1.51 (0.96 to 2.07)	<0.001

Generalized linear models for Waist-height ratio and Systolic blood pressure (Table 3a) and Diastolic blood pressure (Table 3b) with parameters estimates (coefficients) with different adjustment for sex, age and the five health metrics. Model 1: adjusted for sex. Model 2: with adjustment for sex, age and five health metrics. Statistical significance is set at *p* value < 0.05. 95% CI: 95% confidence interval.

a Reported as for a 0.1 increase in WHtR.

WHtR, waist-height ratio; HM, Health Metric; PA, physical activity; HM, health metrics.

In the ROC analysis, WHtR reached an AUC of 0.7285 [95% CI: (0.7203 to 0.7367); *p* < 0.001] in the overall sample, while WHR showed an AUC of 0.6901 (95% CI: 0.6815 to 0.6987; *p* < 0.001) and BMI reached AUC of 0.6869 (95% CI: 0.6783 to 0.6955; *p* < 0.001) (Table 4a). Stratifying by sex, WHtR achieved the highest AUC in females (AUC: 0.7321; 95% CI: 0.7210 to 0.7432; *p* < 0.001) compared to WHR (AUC: 0.6638; 95% CI: 0.6518 to 0.6757; *p* < 0.001) and BMI (AUC: 0.6832; 95% CI: 0.6715 to 0.6950; *p* < 0.001). Similarly, in males, WHtR reached an AUC of 0.6958 (95% CI: 0.6827 to 0.7090; *p* < 0.001) compared to WHR (AUC: 0.6591; 95% CI: 0.6454 to 0.6729; *p* < 0.001) and BMI (AUC: 0.6458; 95% CI: 0.6320 to 0.6595; *p* < 0.001) (Table 4b). WHtR demonstrated superior discriminatory performance compared to both WHR and BMI, as confirmed by DeLong's test for pairwise AUC comparisons in the overall sample (WHtR vs. WHR: ΔAUC: 0.0384, 95% CI: 0.0316 to 0.0452; *p* < 0.001; WHtR vs. BMI: ΔAUC: 0.0415, 95% CI: 0.0358 to 0.0473, *p* < 0.001), and separately in females and males (Supplementary Tables S1a,b). Notably, WHR did not significantly outperform BMI in the overall sample (ΔAUC: −0.0031, 95% CI: −0.0123 to 0.0060; *p* = 0.4996), nor in males (ΔAUC: −0.0133; 95% CI: −0.0274 to 0.0008; *p* = 0.0637), while in females WHR showed a slightly positive difference in AUC compared to BMI (ΔAUC = 0.0195, 95% CI: 0.0060 to 0.0330: *p* = 0.0047). From the AUC comparison between females and males for each index, WHtR and BMI performed significantly better in females than in males, reporting an AUC difference of 0.0362 for WHtR and AUC difference of −0.0375 for BMI (Supplementary Table S2).

**Table 4:** Area under the receiver operating characteristic (ROC) curves.

Table 4a				
Index	AUC	95% CI		*P* value
WHtR	0.7285	(0.7203–0.7367)		<0.001
WHR	0.6901	(0.6815–0.6987)		<0.001
BMI	0.6869	(0.6783–0.6955)		<0.001

Table 4b				
Index	Sex	Area	95% CI	*P* value
WHtR	Female	0.7321	0.7210–0.7432	<0.001
	Male	0.6958	0.6827–0.7090	<0.001
WHR	Female	0.6638	0.6518–0.6757	<0.001
	Male	0.6591	0.6454–0.6729	<0.001
BMI	Female	0.6832	0.6715–0.6950	<0.001
	Male	0.6458	0.6320–0.6595	<0.001

Area Under the Receiver operating characteristic (ROC) curves of waist-height ratio, waist-hip ratio, and BMI for hypertension in the overall sample (Table 4a) and for females and males separately (Table 4b). *P*-values refer to the test of the null hypothesis (Null hypothesis: AUC equals 0.5 = no discrimination).

WHtR, waist-height ratio; WHR, waist hip ratio; BMI, body mass index; AUC, Area under the curve; 95% CI, 95% confidence interval.

## 4 Discussion

We explored the association between WHtR and hypertension, evaluating its discriminatory performance in comparison with BMI and WHR. Our findings confirm a significant, independent association between WHtR and both SBP and DBP, demonstrating that WHtR outperforms both BMI and WHR in identifying individuals suffering from hypertension across both sexes. Participants with abnormal WHtR were predominantly male and older, consistent with the well-established pattern of progressive central fat accumulation with advancing age and the known sex-specific differences in adipose tissue distribution (32); “Comparative Evaluation of Body Mass Index, Waist-Hip Ratio, and Waist-Height Ratio as Predictors of Hypertension in Middle-Aged Individuals,” (33, 34). Visceral adipose tissue directly promotes insulin resistance, endothelial dysfunction, RAAS activation, and sympathetic overactivity, all notorious of hypertension onset and progression (35). These pathways provide a plausible biological mechanism for the association observed in our GLM analysis, although the cross-sectional design does not allow inference on mechanisms. Prospective studies support these findings: a Korean cohort study (36) demonstrated that WHtR may independently predict incident hypertension over a median follow-up of 2.8 years, underscoring the potential longitudinal relevance of this anthropometric index; Wang et al. (*n* = 4,458) divided WHtR into tertiles, reporting that the highest tertile (>0.53) had a 1.45-fold higher hypertension risk vs. the lowest one (≤0.47) and each 0.1 unit WHtR increase raised risk by 30.4% (*p* < 0.001). Furthermore, they confirmed superior diagnostic performance of WHtR over BMI (reported AUC 0.626 vs. 0.607), consistent with our findings (37). BMI does not distinguish between fat mass and lean mass, nor capture the body fat distribution, and lacks sensitivity in detecting central adiposity in individuals with normal body weight but excess central fat, a relevant phenotype for cardiovascular risk (36, 38–41). In a previous analysis from our group, we found a strong association between BMI and hypertension, yet without accounting for fat distribution: the current work shows now that WHtR, capable of capturing central adiposity, may provide superior discriminatory capacity for hypertension (19, 20).

The WHR partially addresses BMI's limitations by accounting for fat distribution and has been shown to have stronger associations with hypertension, compared to BMI, in previous evidence (36, 41). In our ROC analysis, however, WHR did not significantly outperform BMI in the overall sample, but interestingly the AUC for WHR was significantly higher compared to BMI in females, whereas absolute difference was small. This sex-specific pattern could be attributable to hip circumference composition, which partially reflects the gluteofemoral fat depot, not associated with increased cardiometabolic risk (42). Different gluteofemoral fat accumulation between genders may inflate the hip component in WHR, diluting its signal for central adiposity, and reducing its specificity for hypertension risk detection. WHtR, instead, normalizes waist circumference to height alone, exclusively capturing central adiposity. The validated cutoff of 0.5 ensures consistent applicability across sexes, ages, and ethnicities (5, 30). WHtR demonstrated the highest discriminatory accuracy for hypertension in our cohort in the overall sample, confirmed across sexes. DeLong's pairwise comparisons confirmed that WHtR significantly outperformed both BMI and WHR across all groups. These findings align with previous evidence, which identified WHtR as a superior discriminator of cardiometabolic risk compared to BMI and WHR (7, 10). The relatively modest absolute AUC differences need to be contextualised: while the large sample size may accentuate the statistical significance, the 0.04 improvement in AUC provided by WHtR over BMI and WHR is also clinically meaningful. In large screening populations, even small gains in discriminatory performance may potentially translate into clinically meaningful improvements in the identification of at-risk individuals. This translates into potential clinical benefit, offering a reliable anthropometric tool for primary care settings, without any additional costs. Participants with abnormal WHtR exhibited a globally impaired cardiometabolic profile, including higher prevalence of diabetes, hypercholesterolemia, reduced physical activity, lower adherence to healthy diet, consistently with broad cardiometabolic burden associated with central adiposity (9, 43).

Not all evidence supports WHtR as optimal anthropometric predictor of hypertension across all populations: a Japanese study reported that WC in males and BMI in females had stronger associations with blood pressure than WHtR (44); another study carried out on African subjects with high blood pressure, found BMI more strongly correlated with both SBP and DBP than WC or WHtR (45); in paediatric context, a meta-analysis found substantially comparable predictive performance for BMI, WC and WHtR for elevated blood pressure (46). These discordant findings likely reflect heterogeneity in population characteristics, sample size, ethnicity, age distribution, and study design, highlighting that the optimal anthropometric index for hypertension screening may vary by context. A global consensus on this matter requires further large-scale, multiethnic, prospective evidence.

Several limitations of this study deserve mention: the cross-sectional design precludes causal inference and any assessment of temporality between WHtR and hypertension. The inverse association observed between WHtR and likelihood of normotension (with higher WHtR values corresponding to lower probability of normal blood pressure) should therefore be interpreted as a statistical association, not as evidence of a predictive or casual relationship. The predominantly Caucasian cohort limits generalisability of our findings to other populations, as ethnic background and geographical setting may influence central adiposity (47–49). Moreover, the study was conducted in non-clinical setting, including community events in public settings: this also limits generalizability of the findings. Economic, social, environmental, and psychosocial factors may also influence patterns of central adiposity, along with cardio-metabolic thresholds, ultimately impacting on the applicability of a WHtR-based screening strategy (50, 51). Additionally, the specific age distribution of our cohort may not fully reflect a broader demographic, as aging is strongly associated with an un-favourable shift towards central fat deposition (52, 53). Analyses of this study were restricted to participants with complete data, with most exclusions resulted from waist and hip circumferences variables not being collected in the earliest protocol version, a missingness determined by enrolment period and not by individual characteristics. Despite adherence to standardised measurement protocols, the non-clinical community-based assessment setting may have introduced measurement variability for height, waist circumference, and blood pressure. Finally, details on antihypertensive treatment type, intensity and duration were unavailable and could influence blood pressure values independent of anthropometry, representing a potential source of uncontrolled residual confounding, along with other unmeasured clinical factors.

## 5 Conclusions

This study found a significant association between WHtR and arterial hypertension in a large community-dwelling population, reinforcing the potential role of this simple anthropometric measure in cardiovascular risk assessment, specifically for hypertension screening. Elevated WHtR was associated with a globally impaired cardiometabolic health profile, and WHtR demonstrated superior discriminatory performance for hypertension compared to BMI and WHR across both sexes. These findings support the integration of WHtR into routine cardiovascular screening as a simple, low-cost tool, primarily applicable in community-dwelling Caucasian adults. Longitudinal and multiethnic studies are warranted to confirm causal directionality, potential applicability in different settings, ethnicities and socio-economic groups, and evaluate the prognostic value of serial WHtR measurements. Given the cross-sectional nature of these findings, these results should be interpreted as evidence of association, and assertions regarding longitudinal risk prediction should be avoided until further longitudinal data will be provided. Nevertheless, this study contributes evidence supporting WHtR as a practical, anthropometric measure for hypertension screening in community-dwelling subjects, and healthy ageing promotion, pending confirmation in other populations.

## Data Availability

The raw data supporting the conclusions of this article will be made available by the authors, without undue reservation.

## The lookup 8+study group

Department of Geriatrics, Orthopedics and Rheumatology, Università Cattolica del Sacro Cuore, Rome, Italy; Francesco Landi, Roberto Bernabei, Emanuele Marzetti, Riccardo Calvani, Luca Mariotti, Stefano Cacciatore, Hélio José Coelho-Junior, Anna Maria Martone, Gabriele Abbatecola, Clara Agostino, Fiorella Ambrosio, Francesca Banella, Carolina Benvenuto, Maria Modestina Bulla, Caterina Casciani, Camilla Cocchi, Federica Cucinotta, Federica D’Ignazio, Daniele Elmi, Marta Finelli, Francesco Pio Fontanella, Ilaria Gattari, Giordana Gava, Tommaso Giani, Giulia Giordano, Rossella Giordano, Francesca Giovanale, Simone Goracci, Silvia Ialungo, Rosangela Labriola, Elena Levati, Myriam Macaluso, Luca Marrella, Claudia Massaro, Rossella Montenero, Maria Vittoria Notari, Maria Paudice, Martina Persia, Flavia Pirone, Rosa Ragozzino, Carla Recupero, Antonella Risoli, Stefano Rizzo, Daria Romaniello, Giulia Rubini, Barbara Russo, Stefania Satriano, Giulia Savera, Annalise Serra Melechì, Francesca Simeoni, Sofia Simoni, Chiara Taccone, Elena Tagliacozzi, Roberta Terranova, Salvatore Tupputi, Matteo Vaccarella, Emiliano Venditti, Chiara Zanchi, Maria Zuppardo.

Fondazione Policlinico Universitario “Agostino Gemelli” IRCCS, Rome, Italy; Francesco Landi, Emanuele Marzetti, Riccardo Calvani, Luca Mariotti, Stefano Cacciatore, Hélio José Coelho-Junior, Francesca Ciciarello, Vincenzo Galluzzo, Anna Maria Martone, Anna Picca, Andrea Russo, Sara Salini, Matteo Tosato, Damiano Biscotti, Vincenzo Brandi, Lucio Catalano, Camilla Cocchi, Giuseppe Colloca, Emanuela D’Angelo, Mariaelena D’Elia, Federica D’Ignazio, Domenico Fusco, Giordana Gava, Tommaso Giani, Giulia Giordano, Rossella Giordano, Silvia Ialungo, Elena Levati, Myriam Macaluso, Claudia Massaro, Rossella Montenero, Simona Pompei, Elisabetta Serafini, Sofia Simoni.

Department of Medicine and Surgery, LUM University, Casamassima, Italy; Anna Picca.

## References

[1] Mancia G Kreutz R Brunström M Burnier M Grassi G Januszewicz A. 2023 ESH guidelines for the management of arterial hypertension the task force for the management of arterial hypertension of the European society of hypertension. J Hypertens. (2023) 41(12):1874–2071. 10.1097/HJH.0000000000003480 37345492

[2] Zhang H Zhou XD Shapiro MD Lip GYH Tilg H Valenti L. Global burden of metabolic diseases, 1990–2021. Metab Clin Exp. (2024) 160:155999. 10.1016/j.metabol.2024.155999 39151887

[3] McEvoy JW McCarthy CP Bruno RM Brouwers S Canavan MD Ceconi C. 2024 ESC guidelines for the management of elevated blood pressure and hypertension. Eur Heart J. (2024) 45(38):3912–4018. 10.1093/eurheartj/ehae178 39210715

[4] Wang R Liu J Fang G Shi J Zhang C Huang Y. Association between visceral adiposity index and cardiovascular disease: a systematic review and meta-analysis. Nut Metab Cardiovasc Dis. (2025) 35(12):104216. 10.1016/j.numecd.2025.104216 40897610

[5] Moosaie F Fatemi Abhari SM Deravi N Karimi Behnagh A Esteghamati S Dehghani Firouzabadi F. Waist-to-height ratio is a more accurate tool for predicting hypertension than waist-to-hip circumference and BMI in patients with type 2 diabetes: a prospective study. Front Public Health. (2021) 9. 10.3389/fpubh.2021.726288 34692623 PMC8529190

[6] Zhang X Lu X Pan X Shen S Tong N. Role of waist circumference-to-height ratio in assessing adiposity, predicting type 2 diabetes mellitus and other cardiometabolic diseases. Zhong Nan Da Xue Xue Bao Yi Xue Ban J Central South Univ Med Sci. (2024) 49(7):1062–72. 10.11817/j.issn.1672-7347.2024.240259 PMC11495984 39788494

[7] Ashwell M Gunn P Gibson S. Waist-to-height ratio is a better screening tool than waist circumference and BMI for adult cardiometabolic risk factors: systematic review and meta-analysis. Obes Rev. (2012) 13(3):275–86. 10.1111/j.1467-789X.2011.00952.x 22106927

[8] Giordano G Mastrantoni L Calvani R Terranova R D’Ignazio F Risoli A. Nonlinear association between age and verbal fluency and moderation by adiposity: cross-sectional evidence from the look up 8+study. Front Aging Neurosci. (2026) 18. 10.3389/fnagi.2026.1912213 42591501 PMC13461906

[9] Corrêa MM Thumé E De Oliveira ERA Tomasi E. Performance of the waist-to-height ratio in identifying obesity and predicting non-communicable diseases in the elderly population: a systematic literature review. Arch Gerontol Geriatr. (2016) 65:174–82. 10.1016/j.archger.2016.03.021 27061665

[10] Lee CMY Huxley RR Wildman RP Woodward M. Indices of abdominal obesity are better discriminators of cardiovascular risk factors than BMI: a meta-analysis. J Clin Epidemiol. (2008) 61(7):646–53. 10.1016/j.jclinepi.2007.08.012 18359190

[11] Feng Q Bešević J Conroy M Omiyale W Woodward M Lacey B. Waist-to-height ratio and body fat percentage as risk factors for ischemic cardiovascular disease: a prospective cohort study from UK biobank. Am J Clin Nutr. (2024) 119(6):1386–96. 10.1016/j.ajcnut.2024.03.018 38839194 PMC11196863

[12] Lo K Huang YQ Shen G Huang JY Liu L Yu YL. Effects of waist to height ratio, waist circumference, body mass index on the risk of chronic diseases, all-cause, cardiovascular and cancer mortality. Postgrad Med J. (2021) 97(1147):306–11. 10.1136/postgradmedj-2020-137542 32371408

[13] Lu Y Liu S Qiao Y Li G Wu Y Ke C. Waist-to-height ratio, waist circumference, body mass index, waist divided by height0.5 and the risk of cardiometabolic multimorbidity: a national longitudinal cohort study. Nut Metab Cardiovasc Dis. (2021) 31(9):2644–51. 10.1016/j.numecd.2021.05.026 34226121

[14] National Institute for Health and Care Excellence (NICE) (Ed.). Obesity: identification, assessment and management.: Vol. No.189 (2023).

[15] . Savva S Lamnisos D Kafatos A. Predicting cardiometabolic risk: waist-to-height ratio or BMI. A meta-analysis. Diabetes Metab Syndr Obes. (2013) 403:403. 10.2147/DMSO.S34220 PMC3810792 24179379

[16] Nevill AM Brand C Chen R Duncan MJ De C Silveira JF Sehn AP. NICE waist-to-height ratio guidelines “to have a waist< 0.5 of your height”, are suitable for children but misleading for adolescents. Nut Metab Cardiovasc Dis. (2025) 35(10):104117. 10.1016/j.numecd.2025.104117 40451675

[17] Jayedi A Rashidy-Pour A Khorshidi M Shab-Bidar S. Body mass index, abdominal adiposity, weight gain and risk of developing hypertension: a systematic review and dose–response meta-analysis of more than 2.3 million participants. Obes Rev. (2018) 19(5):654–67. 10.1111/obr.12656 29334692

[18] Giordano G Mastrantoni L Landi F. Development and validation of quantile regression forests for prediction of reference quantiles in handgrip and chair-stand test. J Cachexia Sarcopenia Muscle. (2025) 16(3). 10.1002/jcsm.13868 40525650 PMC12171994

[19] Landi F Calvani R Picca A Tosato M D’Angelo E Martone AM. Relationship between cardiovascular health metrics and physical performance in community-living people: results from the longevity check-up (lookup) 7+project. Sci Rep. (2018) 8(1):16353. 10.1038/s41598-018-34746-4 30397361 PMC6218517

[20] Landi F Calvani R Picca A Tosato M Martone AM Ortolani E. Body mass index is strongly associated with hypertension: results from the longevity check-up 7+study. Nutrients. (2018) 10(12):1976. 10.3390/nu10121976 30551656 PMC6316192

[21] Von Elm E Altman DG Egger M Pocock SJ Gøtzsche PC Vandenbroucke JP. The strengthening the reporting of observational studies in epidemiology (STROBE) statement. Epidemiology. (2007) 18(6):800–4. 10.1097/EDE.0b013e3181577654 18049194

[22] Cacciatore S Martone AM Ciciarello F Galluzzo V Gava G Massaro C. Waist-to-calf circumference ratio as a potential indicator of diabetes risk: results from the longevity check-up (lookup) 8+. Sci Rep. (2024) 14(1):28882. 10.1038/s41598-024-79329-8 39572573 PMC11582644

[23] Cacciatore S Calvani R Mancini J Ciciarello F Galluzzo V Tosato M. Poor sleep quality is associated with probable sarcopenia in community-dwelling older adults: results from the longevity check-up (lookup) 8+. Exp Gerontol. (2025) 200:112666. 10.1016/j.exger.2024.112666 39709067

[24] Landi F Calvani R Tosato M Martone AM Fusco D Sisto A. Age-related variations of muscle mass, strength, and physical performance in community-dwellers: results from the Milan EXPO survey. J Am Med Dir Assoc. (2017) 18(1):88.e17–24. 10.1016/j.jamda.2016.10.007 27914849

[25] Coelho-Júnior HJ Calvani R Picca A Tosato M Landi F Marzetti E. Engagement in aerobic exercise is associated with a reduced prevalence of sarcopenia and severe sarcopenia in Italian older adults. J Pers Med. (2023) 13(4):655. 10.3390/jpm13040655 PMC10144721 37109041

[26] Rapi S Bazzini C Tozzetti C Sbolci V Modesti PA. Point-of-care testing of cholesterol and triglycerides for epidemiologic studies: evaluation of the multicare-in system. Transl Res. (2009) 153(2):71–6. 10.1016/j.trsl.2008.11.010 19138651

[27] ElSayed NA Aleppo G Bannuru RR Bruemmer D Collins BS Ekhlaspour L. 2. Diagnosis and classification of diabetes: standards of care in diabetes—2024. Diabetes Care. (2024) 47(Supplement_1):S20–42. 10.2337/dc24-S002 38078589 PMC10725812

[28] Hayman LL Martyn-Nemeth P. A new metric for promoting cardiovascular health: life’s essential 8. J Cardiovasc Nur. (2022) 37(6):509–11. 10.1097/JCN.0000000000000946 PMC9665048 36037296

[29] Ness-Abramof R Apovian CM. Waist circumference measurement in clinical practice. Nutr Clin Pract. (2008) 23(4):397–404. 10.1177/0884533608321700 18682591

[30] Browning LM Hsieh SD Ashwell M. A systematic review of waist-to-height ratio as a screening tool for the prediction of cardiovascular disease and diabetes: 0·5 could be a suitable global boundary value. Nutr Res Rev. (2010) 23(2):247–69. 10.1017/S0954422410000144 20819243

[31] . DeLong ER DeLong DM Clarke-Pearson DL. Comparing the areas under two or more correlated receiver operating characteristic curves: a nonparametric approach. Biometrics. (1988) 44:837. 10.2307/2531595 3203132

[32] Balkau B Sapinho D Petrella A Mhamdi L Cailleau M Arondel D. Prescreening tools for diabetes and obesity-associated dyslipidaemia: comparing BMI, waist and waist hip ratio. The D.E.S.I.R. Study. Eur J Clin Nutr. (2006) 60(3):295–304. 10.1038/sj.ejcn.1602308 16278693 PMC2065791

[33] Comparative Evaluation of Body Mass Index. Waist-hip ratio, and waist-height ratio as predictors of hypertension in middle-aged individuals. J Heart Valve Dis. (2025) 30(6):61–4.

[34] Wang W Wang K Li T Xiang H Ma L Fu Z. A discussion on utility and purposed value of obesity and abdomen obesity when body mass index, waist circumference, waist to hip ratio used as indexes predicting hypertension and hyper-blood glucose. Zhonghua Liu Xing Bing Xue Za Zhi. (2002) 23(1):16–9. 12015102

[35] Mathieu P Poirier P Pibarot P Lemieux I Després J-P. Visceral obesity. Hypertension. (2009) 53(4):577–84. 10.1161/HYPERTENSIONAHA.108.110320 19237685

[36] Choi JR Koh SB Choi E. Waist-to-height ratio index for predicting incidences of hypertension: the ARIRANG study. BMC Public Health. (2018) 18(1):767. 10.1186/s12889-018-5662-8 29921256 PMC6008942

[37] Wang Z Shi Q Yan X Tang J Zhang J. There was a similar U-shaped nonlinear association between waist-to-height ratio and the risk of new-onset hypertension: findings from the CHNS. Front Nutr. (2023) 10. 10.3389/fnut.2023.1304521 PMC10752934 38156282

[38] Chung W Park CG Ryu O-H. Association of a new measure of obesity with hypertension and health-related quality of life. PLoS One. (2016) 11(5):e0155399. 10.1371/journal.pone.0155399 27183311 PMC4868302

[39] Heymsfield SB Scherzer R Pietrobelli A Lewis CE Grunfeld C. Body mass index as a phenotypic expression of adiposity: quantitative contribution of muscularity in a population-based sample. Int J Obes. (2009) 33(12):1363–73. 10.1038/ijo.2009.184 PMC3156622 19773739

[40] Oreopoulos A Padwal R McAlister FA Ezekowitz J Sharma AM Kalantar-Zadeh K. Association between obesity and health-related quality of life in patients with coronary artery disease. Int J Obes. (2010) 34(9):1434–41. 10.1038/ijo.2010.73 20386551

[41] Son YJ Kim J Park H-J Park SE Park C-Y Lee W-Y. Association of waist-height ratio with diabetes risk: a 4-year longitudinal retrospective study. Endocrinol Metab. (2016) 31(1):127. 10.3803/EnM.2016.31.1.127 PMC4803548 26754587

[42] Manolopoulos KN Karpe F Frayn KN. Gluteofemoral body fat as a determinant of metabolic health. Int J Obes. (2010) 34(6):949–59. 10.1038/ijo.2009.286 20065965

[43] Ashwell M Mayhew L Richardson J Rickayzen B. Waist-to-height ratio is more predictive of years of life lost than body mass index. PLoS One. (2014) 9(9):e103483. 10.1371/journal.pone.0103483 25198730 PMC4157748

[44] Sakurai M Miura K Takamura T Ota T Ishizaki M Morikawa Y. Gender differences in the association between anthropometric indices of obesity and blood pressure in Japanese. Hypertens Res. (2006) 29(2):75–80. 10.1291/hypres.29.75 16755140

[45] Adeoye AM Adebayo O Nwosu M Adebiyi A Owolabi MO Tayo BO. Assessment of measures of adiposity that correlate with blood pressure among hypertensive Africans. Ann Ib Postgrad Med. (2017) 15(2):82–7. 29556161 PMC5846179

[46] Tao J-M Wei W Ma X-Y Huo Y-X Hu M-D Li X-F. Diagnostic accuracy of anthropometric indices for discriminating elevated blood pressure in pediatric population: a systematic review and a meta-analysis. BMC Pediatr. (2022) 22(1):19. 10.1186/s12887-021-03062-8 34983442 PMC8725266

[47] Higgins V Nazroo J Brown M. Pathways to ethnic differences in obesity: the role of migration, culture and socio-economic position in the UK. SSM Popul Health. (2019) 7:100394. 10.1016/j.ssmph.2019.100394 31032393 PMC6477158

[48] Lear SA Humphries KH Kohli S Chockalingam A Frohlich JJ Birmingham CL. Visceral adipose tissue accumulation differs according to ethnic background: results of the multicultural community health assessment trial (M-CHAT). Am J Clin Nutr. (2007) 86(2):353–9. 10.1093/ajcn/86.2.353 17684205

[49] Williams R Periasamy M. Genetic and environmental factors contributing to visceral adiposity in Asian populations. Endocrinol Metab. (2020) 35(4):681–95. 10.3803/EnM.2020.772 PMC7803598 33397033

[50] Capoccia D Milani I Colangeli L Parrotta ME Leonetti F Guglielmi V. Social, cultural and ethnic determinants of obesity: from pathogenesis to treatment. Nut Metab Cardiovasc Dis. (2025) 35(6):103901. 10.1016/j.numecd.2025.103901 40087047

[51] Nazare J-A Smith JD Borel A-L Haffner SM Balkau B Ross R. Ethnic influences on the relations between abdominal subcutaneous and visceral adiposity, liver fat, and cardiometabolic risk profile: the international study of prediction of intra-abdominal adiposity and its relationship with cardiometabolic risk/intra-abdominal adiposity. Am J Clin Nutr. (2012) 96(4):714–26. 10.3945/ajcn.112.035758 22932278

[52] Hunter GR Gower BA Kane BL. Age related shift in visceral fat. Int J Body Compos Res. (2010) 8(3):103–8. 24834015 PMC4018766

[53] Kuk JL Lee S Heymsfield SB Ross R. Waist circumference and abdominal adipose tissue distribution: influence of age and sex. Am J Clin Nutr. (2005) 81(6):1330–4. 10.1093/ajcn/81.6.1330 15941883

