# Supplementary material for: Association between waist-height ratio and hypertension: results from the lookup 8+ study
Source: Front Med (Lausanne). 2026 Oct 21;13:1975590. doi: 10.3389/fmed.2026.1975590 (PMC13731364; doi:10.3389/fmed.2026.1975590)
Supplement: Supplementary file 1 [file Supplementaryfile1.docx]

**Supplemental material**

**Supplemental Figure 1: Flow diagram of participants selection**

Excluded patients with missing data in Blood Pressure, Age, Sex:

895

Total patients recruited from June 1^st^ 2015,
to December 1^st^ 2025:

24,445

Excluded patients with missing data in Waist, Height. Hip Measurements:

8,297

Total patients
at this stage:

23,550

Excluded patients with missing data in all other variables:

1,108

Total patients
at this stage:

15,523

Total patients included in the analysis:

14,415

**Supplementary Figure 1.** Flow diagram of participants exclusion based on missing data in the variables of interest for every step.

**Supplemental table 1: Pairwise AUC comparisons**

**Table S1a**

| **Comparison** | **AUC difference** | **95% CI** | **P value** |
| --- | --- | --- | --- |
| **Waist-height ratio VS Waist-hip ratio** | 0.0384 | (0.0316 to 0.0452) | <0.001 |
| **Waist-height ratio VS BMI** | 0.0415 | (0.0358 to 0.0473) | <0.001 |
| **Waist-hip ratio VS BMI** | -0.0031 | (-0.0123 to 0.0060) | 0.4996 |

**Table S1b**

| **Variable(s)** | **Sex** | **AUC difference** | **95% CI** | **P value** |
| --- | --- | --- | --- | --- |
| **Waist- height ratio VS**  **Waist-hip ratio** | Female | 0.0683 | 0.0592 to 0.0775 | <0.001 |
|  | Male | 0.0367 | 0.0270 to 0.0464 | <0.001 |
| **Waist-height ratio VS**  **BMI** | Female | 0.0488 | 0.0410 to 0.0566 | <0.001 |
|  | Male | 0.0500 | 0.0407 to 0.0594 | <0.001 |
| **Waist-hip ratio VS**  **BMI** | Female | 0.0195 | 0.0060 to 0.0330 | 0.0047 |
|  | Male | -0.0133 | -0.0274 to 0.0008 | 0.0637 |

**Table S1.** Pairwise AUC Comparisons (DeLong's Test) for AUC in the overall sample (Table S1a) and in Females and Males (Table S1b).

BMI: body mass index; AUC: Area under the curve; 95% CI: 95% confidence interval.

**Supplemental table 2: AUC comparison in females and males**

| **Index** | **AUC male** | **AUC female** | **AUC_diff** | **95% CI** | **P value** |
| --- | --- | --- | --- | --- | --- |
| **WHtR** | 0.6958 | 0.7321 | -0.0362 | (-0.0534 to -0.0190) | <0.001 |
| **WHR** | 0.6591 | 0.6638 | -0.0046 | (-0.0229 to 0.0136) | 0.6175 |
| **BMI** | 0.6458 | 0.6832 | -0.0375 | (-0.0556 to -0.0193) | <0.001 |

Table 2 AUC Comparisons (DeLong's Test) for WHtR, WHR and BMI in Females and Males.

WHtR: Waist-height ratio; WHR: Waist-hip ratio; BMI: body mass index; AUC: Area under the curve; 95% CI: 95% confidence interval.

**Strobe checklist**

STROBE Statement- checklist of items that should be included in reports of observational studies

|  | **Item No** | **Recommendation** | **Page No** |
| --- | --- | --- | --- |
| **Title and abstract** | 1 | (*a*) Indicate the study’s design with a commonly used term in the title or the abstract | 1 |
|  |  | (*b*) Provide in the abstract an informative and balanced summary of what was done and what was found | 2 |
| **Introduction** | | | |
| Background/rationale | 2 | Explain the scientific background and rationale for the investigation being reported | 3-4 |
| Objectives | 3 | State specific objectives, including any prespecified hypotheses | 4 |
| **Methods** | | | |
| Study design | 4 | Present key elements of study design early in the paper | 5 |
| Setting | 5 | Describe the setting, locations, and relevant dates, including periods of recruitment, exposure, follow-up, and data collection | 5-6 |
| Participants | 6 | (*a*) Give the eligibility criteria, and the sources and methods of selection of participants | 5 |
| Variables | 7 | Clearly define all outcomes, exposures, predictors, potential confounders, and effect modifiers. Give diagnostic criteria, if applicable | 5-6 |
| Data sources/ measurement | 8* | For each variable of interest, give sources of data and details of methods of assessment (measurement). Describe comparability of assessment methods if there is more than one group | 5-7 |
| Bias | 9 | Describe any efforts to address potential sources of bias | 13 |
| Study size | 10 | Explain how the study size was arrived at | NA |
| Quantitative variables | 11 | Explain how quantitative variables were handled in the analyses. If applicable, describe which groupings were chosen and why | 6-7 |
| Statistical methods | 12 | (*a*) Describe all statistical methods, including those used to control for confounding | 7-8 |
|  |  | (*b*) Describe any methods used to examine subgroups and interactions | 8 |
|  |  | (*c*) Explain how missing data were addressed | 8 |
|  |  | (*d*) If applicable, describe analytical methods taking account of sampling strategy | 8 |
|  |  | (*e*) Describe any sensitivity analyses | NA |
| **Results** | | | |
| Participants | 13* | (a) Report numbers of individuals at each stage of study-eg numbers potentially eligible, examined for eligibility, confirmed eligible, included in the study, completing follow-up, and analysed | Supplemental material |
|  |  | (b) Give reasons for non-participation at each stage | Supplemental  material |
|  |  | (c) Consider use of a flow diagram | Supplemental material |
| Descriptive data | 14* | (a) Give characteristics of study participants (eg demographic, clinical, social) and information on exposures and potential confounders | 9-10 |
|  |  | (b) Indicate number of participants with missing data for each variable of interest | NA |
| Outcome data | 15* | Report numbers of outcome events or summary measures | 9-10 |
| Main results | 16 | (*a*) Give unadjusted estimates and, if applicable, confounder-adjusted estimates and their precision (eg, 95% confidence interval). Make clear which confounders were adjusted for and why they were included | 9-10 |
|  |  | (*b*) Report category boundaries when continuous variables were categorized | 9-10 |
|  |  | (*c*) If relevant, consider translating estimates of relative risk into absolute risk for a meaningful time period | NA |
| Other analyses | 17 | Report other analyses done—eg analyses of subgroups and interactions, and sensitivity analyses | 9-10 |
| **Discussion** | | | |
| Key results | 18 | Summarise key results with reference to study objectives | 11-13 |
| Limitations | 19 | Discuss limitations of the study, taking into account sources of potential bias or imprecision. Discuss both direction and magnitude of any potential bias | 13 |
| Interpretation | 20 | Give a cautious overall interpretation of results considering objectives, limitations, multiplicity of analyses, results from similar studies, and other relevant evidence | 11-13 |
| Generalisability | 21 | Discuss the generalisability (external validity) of the study results | 14 |
| **Other information** | | | |
| Funding | 22 | Give the source of funding and the role of the funders for the present study and, if applicable, for the original study on which the present article is based | 15 |
